# A new class of large band gap quantum spin hall insulators: 2D fluorinated group-IV binary compounds

**DOI:** 10.1038/srep26123

**Published:** 2016-05-23

**Authors:** J. E. Padilha, R. B. Pontes, T. M. Schmidt, R. H. Miwa, A. Fazzio

**Affiliations:** 1 Universidade Federal do Paraná, Campus Avançado de Jandaia do Sul, Jandaia do Sul, PR, Brazil; 2 Instituto de Física, Universidade Federal de Goiás, 74690-900, Goiânia, GO, Brazil; 3 Instituto de Física, Universidade Federal de Uberlândia, Uberlândia, MG, Brazil; 4 Centro de Ciências Naturais e Humanas, Universidade Federal do ABC, Santo André, São Paulo, Brazil 09210-170; 5 Instituto de Física, Universidade de São Paulo, CP 66318, 05315-970, São Paulo, SP, Brazil

## Abstract

We predict a new class of large band gap quantum spin Hall insulators, the fluorinated PbX (X = C, Si, Ge and Sn) compounds, that are mechanically stable two-dimensional materials. Based on first principles calculations we find that, while the PbX systems are not topological insulators, all fluorinated PbX (PbXF_2_) compounds are 2D topological insulators. The quantum spin Hall insulating phase was confirmed by the explicitly calculation of the Z_2_ invariant. In addition we performed a thorough investigation of the role played by the (i) fluorine saturation, (ii) crystal field, and (iii) spin-orbital coupling in PbXF_2_. By considering nanoribbon structures, we verify the appearance of a pair of topologically protected Dirac-like edge states connecting the conduction and valence bands. The insulating phase which is a result of the spin orbit interaction, reveals that this new class of two dimensional materials present exceptional nontrivial band gaps, reaching values up to 0.99 eV at the Γ point, and an indirect band gap of 0.77 eV. The topological phase is arisen without any external field, making this system promising for nanoscale applications, using topological properties.

Topological insulators (TIs) have been the subject of numerous studies addressing not only their applications, but also the fundamental physics behind their electronic properties. Two-dimensional (2D) TI is characterised by the presence of a non-trivial (bulk) energy gap, and the appearance of topologically protected (edge) states upon its contact with a trivial insulator^1^. Those topologically protected states are composed by 1D metallic channels, with wave vector parallel to the edge sites, being protected by the time reversal symmetry. In this case, backscattering processes are fully prohibited, and thus promoting dissipationless electronic currents.

One year after its successful synthesis^2^, graphene was the first theoretical proposal of a 2D TI^3^. However, due to the weak spin-orbital coupling (SOC) of the carbon atoms, the topological phase in graphene is not experimentally observable^4^,^5^. Similarly to graphene, other group-IV sheets have been recently proposed, and the investigations pointed out that hexagonal lattices of buckled silicon or germanium atoms (silicene and germanene) also present a topological phase, by opening an energy gap mediated by the SOC^6^,^7^. Further studies indicate that such a topological phase can be tuned by an external electric field^8^,^9^,^10^. Silicene presents a strong technological appeal and indeed it has been successfully synthesized^11^ on solid surfaces. Upon hydrogen functionalization in silicene (silicane)^12^,^13^,^14^ as well as in germanene (germanane)^15^, the topological gap at the K and K′ points has been suppressed, and those systems become trivial insulators. In a recent study, the formation of topologically protected states along the edge sites of germanene/germanane has been proposed, namely a topological/trivial interface structure^16^, where the protected edge states were well characterized.

The appearance of a non-trivial band gap in TIs is ruled by a suitable synergy between the crystal-field and the SOC. Large non-trivial bulk band gap is quite desirable, since it allows operations at room temperatures. Here, atomistic simulations, based on *ab initio* methods, play an important role, (i) electronic structure calculations may provide a possible route to get non-trivial band gaps, and (ii) informations with respect to the energetic stability can be obtained through total energy calculations. The latter [(ii)] is very important to the experimental realization of those proposed new materials. Indeed, in a recent theoretical work^17^, a large non-trivial band gap (~0.3 eV) was predicted for functionalized tin monolayer (stanene). Few years latter, a single layer of Sn-film was successfully synthesized^18^.

Further theoretical studies suggest a trivial → non-trivial phase transition upon external strain in a single layer of functionalized germanene^19^. Similarly for group V compounds like Sb^20^; and group III-V combinations as BBi, AlBi^21^, and GaAs^22^. Meanwhile, by making a suitable combination of elements with large SOC, *e.g.* like TlBi^23^, the 2D TI phase may appear at the (fully relaxed) equilibrium geometry without any external agent like strain or electric fields. In this case, we can infer that 2D systems composed by lead is quite interesting. Indeed, in ref. 24 the authors verified that Pb atoms forming a hexagonal buckled lattice, like its counterpart silicene, is a 2D TI with large energy gap. However, recent studies, based on the same calculation approach, indicate that functionalized Pb*X (X* = H, F, Cl, Br, and I) compounds are dynamically unstable^19^.

In this letter we present a new class of large band gap quantum spin Hall insulators (QSHIs) composed by a fully fluorinated binary compounds, PbXF_2_ (X = C, Si, Ge, and Sn). By computing the phonon frequencies we verified that those materials are mechanically stable. Our first principles results shows that all PBXF_2_ are 2D TIs based on the explicitly calculation of Z_2_ invariant. This group-IV compounds present quite large band gaps, reaching up to 0.77 eV, more than two times the largest band gap for a two dimensional material that has been experimentally obtained, the 2D TI stanene^17^,^18^. Furthermore, no external effect, like strain, is necessary in these materials to reach the nontrivial phase. Finally by considering nanoribbon structures, we verify the formation of spin-locked 1D Dirac-like edge states, providing further support the nontrivial phase of PbXF_2_.

## Results

**Figure 1:**
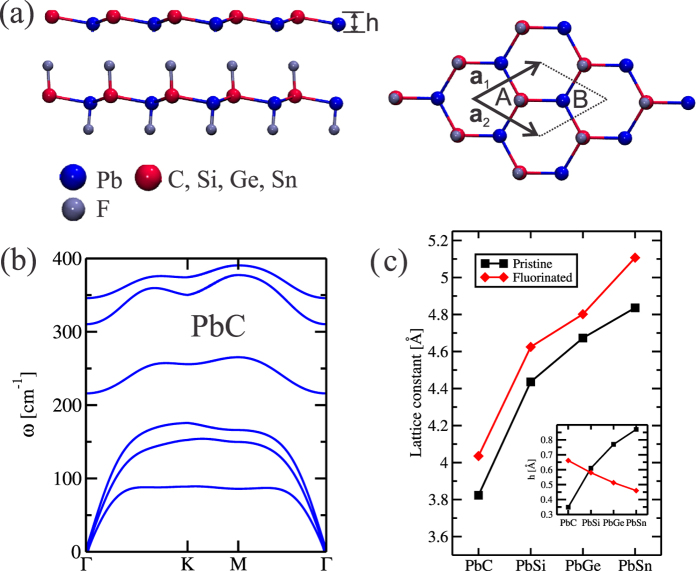
(**a**) Ball stick model of the hexagonal binary lead compounds PbX (X = C, Si, Ge and Sn) (Left: side view of the non-fluorinated (top) and fluorinated (bottom) structure showing the buckling height; Right: Top view showing the unit cell and lattice vectors of the system). (**b**) Phonon spectra for the non-fluorinated PbC material. (**c**) Evolution of the lattice constant for each system in both situations: non-fluorinated (black squares) and fluorinated (red diamonds). The inset stands for the buckling height (h), in Å, as a function of the investigated structures, with (red line) and without (black line) fluorine atoms.

**Figure 2:**
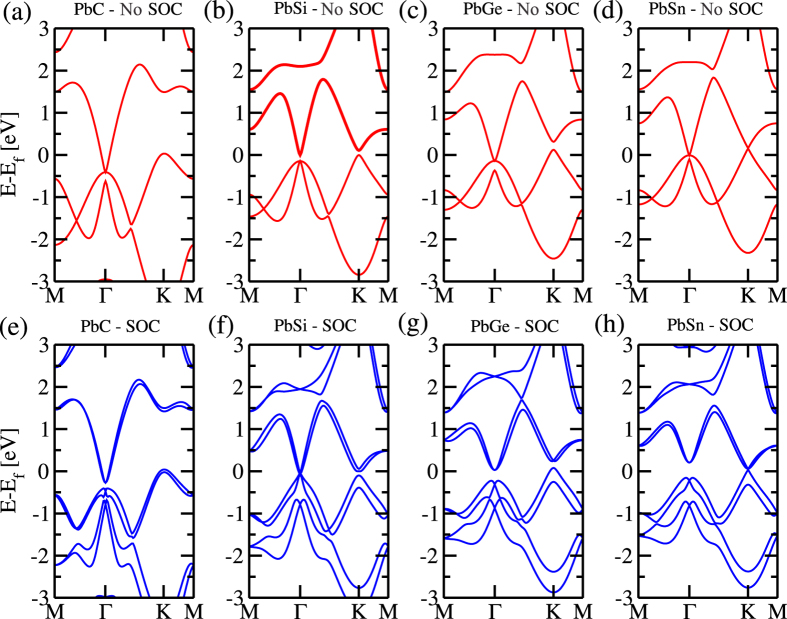
DFT-calculated electronic band structure for the PbX materials without (**a–e**) and with (**f–h**) spin-orbit coupling.

**Figure 3:**
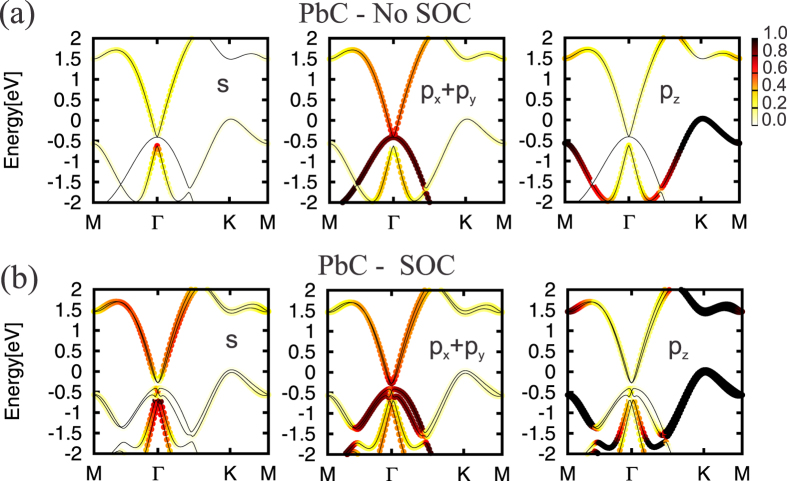
Orbital resolved band structure for the PbC material (**a**) with and (**b**) without spin orbit coupling. In each figure on the left we present the *s* states, on the middle the *p*_*x*_ + *p*_*y*_ states and on the right side the *p*_*z*_ orbitals.

The PbX structure is composed by a buckled hexagonal lattice, with Pb and X atoms on different planes [upper panel of Fig. 1(a)]. Here, the mechanical stability of those PbX (X = C, Si, Ge, and Sn) systems was examined through the calculation of the phonon spectra. In Fig. 1(b) we show the phonon spectra of PbC, there are no imaginary frequencies, and thus confirming its mechanical stability. The same behavior was verified for all other structures, including the fluorinated ones (PbXF_2_). In this work we only considered the PbX systems with fluorine atoms (See supplementary material). The PbX structures present a semimetallic character, with the presence of partially occupied states near the Γ and K points, as depicted in Fig. 2. By projecting the *s* and *p* orbital contributions on the band structure, we show that the electronic states at the Γ and K points, near the Fermi level, are mostly composed by *p*_*x*_/*p*_*y*_ (*σ*) and *p*_*z*_ (*π*) orbitals, as shown for PbC in Fig. 3(a). The inclusion of SO interactions [Fig. 2(e–h)] induces energy splittings on the electronic structure, however, the (semi)metallic character of the systems have been maintained. At the Γ point, the SO couplings splits the partially occupied *p*_*x*_ and *p*_*y*_ orbitals, while the *p*_*z*_ states are kept partially occupied around the K point, Fig. 3(b).

We may have a semiconductor PbX system upon the passivation of those (partially occupied) *p*_*z*_ orbitals. Indeed, here we use fluorine to passivate the dangling bonds, forming the PbXF_2_ structure [lower panel of Fig. 1(a)]. The adsorption of fluorine atoms in PbX (PbX → PbXF_2_) is an exothermic process. By comparing the total energies of the separated components, namely, PbX and an isolated F_2_ molecule with the total energy of the final system, PbXF_2_; we find an energy gain of ~1.5 eV per F-atom. The lattice parameters of the PbX as well the PbXF_2_ structures increase with the covalent radius of the X element, as shown in Fig. 1(c). The buckling height (h) for the pristine PbX also increases from X = C to X = Sn (see inset in Fig. 1(c)), following a competition between 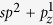 and *sp*^3^ hybridization. The same 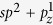 and *sp*^3^ competition rules the equilibrium geometry of fluorinated PbXF_2_ systems. However, the difference on the electronegativity between the X and fluorine atoms plays an important role; where such a difference increases from C to Sn. Here, we verify that the *sp*^3^ character becomes stronger (weaker) in PbCF_2_ (PbSnF_2_). Indeed, the Sn-5p_*z*_ orbital of PbSnF_2_ becomes nearly empty, promoting a reduction on the buckling height; in contrast our total charge density calculations, for the PbCF_2_ system, reveal the covalent character along the C-F chemical bonds. In addition, further atomic relaxations take place, increasing the lattice constant of PbXF_2_, in order to minimize the strain energy of the fluorinated systems.

**Figure 4:**
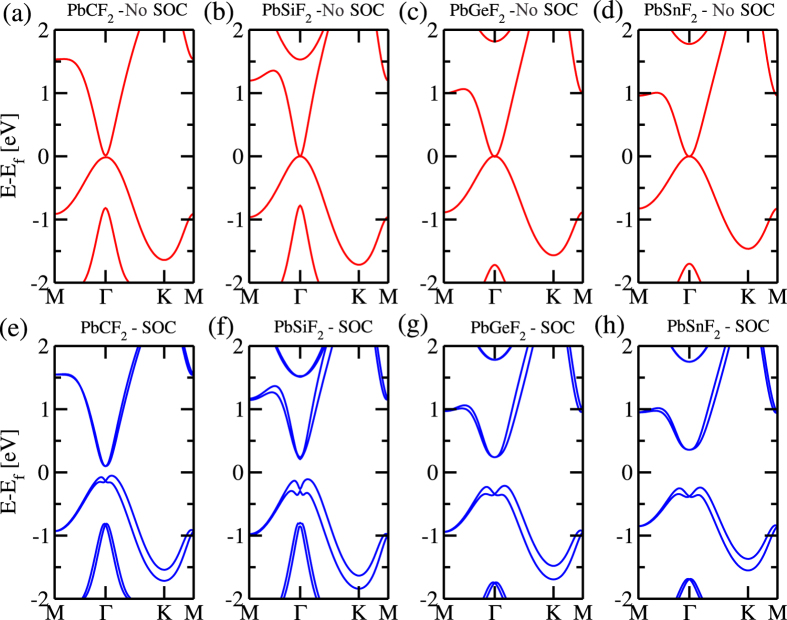
DFT-calculated electronic band structure for the PbXF_2_ materials without (**a–e**) and with (**f–h**) spin-orbit coupling.

**Figure 5:**
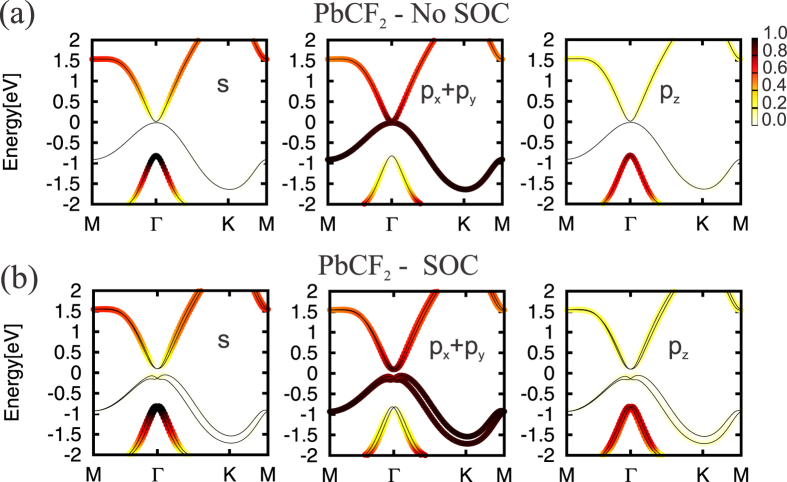
Orbital resolved band structure for the PbCF_2_ material (**a**) with and (**b**) without spin orbit coupling. In each figure on the left we present the *s* states, on the middle the *p*_*x*_ + *p*_*y*_ states and on the right side the *p*_*z*_ orbitals.

The effect of fluorine passivation in the band structure is shown in Fig. 4(a–d); the metallic bands near the K-point are washed out, however, the absence of energy gap at the Γ-point has been maintained. Similarly to the unpassivated PbX systems, those partially occupied states are composed *p*_*x*_ and *p*_*y*_ orbitals. Further inclusion of the SO interaction, the PbXF_2_ sheets present an energy gap near the Γ point, as shown in Fig. 4(e–h). In Fig. 5(a,b) we present the orbital resolved electronic band structure of PbCF_2_ with and without the SOC; where we shown (i) the energy gap at the Γ-point (composed of *p*_*x*_ and *p*_*y*_); (ii) the last but one occupied is a mainly *s* orbital with some hybridization with *p*_*z*_ orbitals that comes from the Pb-X bonds; and (iii) there are no partially *p*_*z*_ orbitals near the K-point. The same picture [(i)–(iii) above] has been verified for the other PbXF_2_ (X = Si, Ge, and Sn) systems. It is worth noting that the (partial) passivation of the dangling bonds of those 2D systems can be done upon their adsorption on suitable solid surfaces^25^.

**Figure 6:**
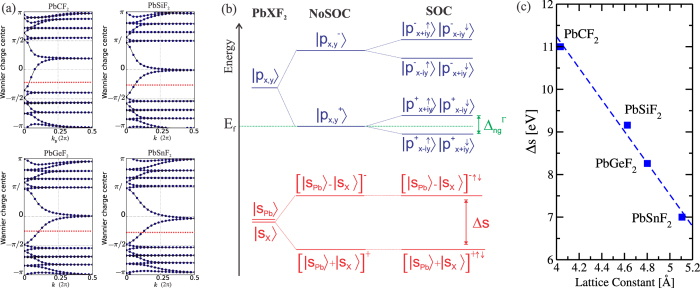
(**a**) Tracking of the evolution of the Wannier Charge Centers (WCCs) between two TRIM points in the reciprocal plane *k*_*z*_ = 0. In dashed red we have a reference line to track the number of Wannier center pair switching in half of the Brillouin Zone. (**b**) Schematic representation of the evolution of the s_*X*;*Pb*_ and p_*x*;*y*_ orbitals into the conduction and valence bands at the Γ point for PbXF_2_ systems, with and without the inclusion of the spin-orbital coupling in the calculations. (**c**) Split of the s orbital (Δs), in eV, as a function of the lattice constant of the PbXF_2_ structure.

The topological nature of PbXF_2_ can be confirmed by a nonzero topological invariant Z_2_. To determine the Z_2_ invariant the WCCs method were used. The topological invariant Z_2_ = 1 were obtained for all PbXF_2_ systems, as depicted in Fig. 6(a) by the evolution of the WCCs between two time-reversal invariant momenta (TRIM) of the Brillouin zone. We can see that the WCCs always cross the red dashed line odd times, giving Z_2_ = 1. On the other hand, we may also verify the topological nature of PbXF_2_ based on our electronic band structure results. Indeed, in Fig. 6(b) we present an schematic energy diagram of PbXF_2_, for the electronic states near the Fermi level, based upon the statements (i)–(iii) from Fig. 5. There is no inversion symmetry, and the total *s* state is a combination of *s*_*Pb*_ and *s*_*X*_. The bonding (+) and antibonding (−) states are labeled [|*S*_*Pb*_〉 ± |*S*_*X*_〉]^±^. The top of valence band and the bottom of the conduction band also comes from the Pb-X bonds and they are degenerated bonding states |*P*_*x*,*y*_〉^+^ and due to the split by SO interactions, a band gap is opened. The compound will be in a nontrivial phase, while the antibonding [|*S*_*Pb*_〉 − |*S*_*X*_〉]^−^ state is still inside the valence band, as schematically shown in Fig. 6(b). The splitting between the bonding and antibonding state, Δ*S*, is mediated by the crystal field of PbXF_2_, and it decreases from X = C to X = Sn, as shown in Fig. 6(c). The decreasing of Δ*S* follows the Pb-X bondlengths, as stronger is the Pb-X interactions, stronger will be the splitting. Also from Fig. 4(e–h), we can see that the antibonding *s* state goes deeper inside the valence band as the PbXF_2_ lattice parameter increases.

**Figure 7:**
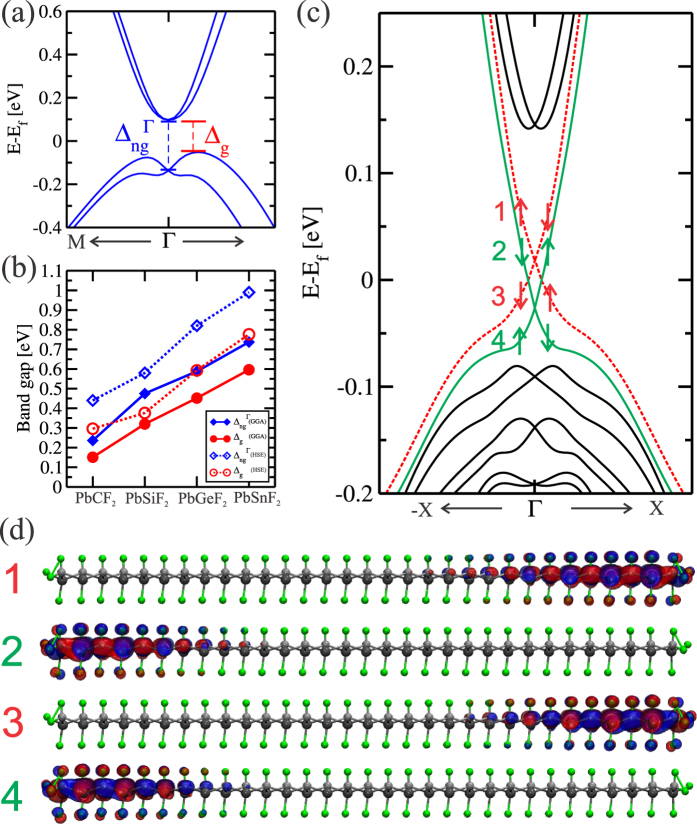
(**a**) Electronic band structure for PbCF_2_ around the fermi level. 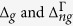 stand for the efective band gap of the material and the non-trivial band gap opened at the Γ point due to the spin orbit coupling, respectively. (**b**) Evolution of the band gaps(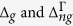) as function of the composition of the fluorinated materials determined with GGA-PBE and HSE-06. (**c**) Electronic band structure for a armchair nanoribbon representing its edge states together with its spin polarization. (**d**) Wave function for a *k*–*point* close to the Γ point for each edge state presented in (**c**).

It is worth noting that the lowest unoccupied and the highest occupied |*P*_*x*,*y*_〉^+^ states of PbXF_2_ present a Rashba-splitting, as shown Fig. 7(a) for PbCF_2_. This effect turns the fluorinated 2D systems with direct band gap at the Γ point (Δ_*ng*_), but a global indirect band gap (Δ_*g*_) [Fig. 7(a)]. It is well known that GGA approximation underestimates the band gap, we thus also carried the band gap calculations with hybrid functional HSE06^26^,^27^. Depicted in Fig. 7(b) we present the results for the band gap calculated with GGA (filled shapes) and with HSE-06 (empty shapes). (The band structure for all PbXF_2_ systems with SOC and HSE-06 are presented in Supplementary Information). One can see that the band gap of the materials are enhanced, reaching values around 0.99 eV. In both approximation (GGA and HSE-06), the band gap always increases from X = C to X = Sn. The value of the band gap of a topological insulator is directly related to the strength of the SO interaction, that in our case must increase for heavy X atoms. For the PbXF_2_ materials, both band gaps increases following the atomic number (Z) of the X atomic element, presented in Fig. 7(b). Another point is that the decoration of the PbX system with F tends to decrease the buckling height, increasing the lattice constant, the result if a external strain were applied to the material, and this effect alto increases the band gap of the materials^17^,^19^,^22^. Most interesting is that we can have a 2D TI with a direct band gap at Γ close to 1 eV, and a global band gap of 0.75 eV for the PbSnF_2_. This huge 2D TI band gap, make this system not only experimentally feasible for the observation of the QSHE at room temperature, but also for applications in 2D nanodevices, using topological properties.

One key feature presented by a 2D topological insulator is the existence of an odd number of topologically protected helical Dirac-like edge states, connecting the conduction and valence bands when we contact the 2D TI to a trivial insulator. To this end we construct nanoribbons (NRs) for each PbXF_2_ compound. In Fig. 7(c,d) we present the Dirac-like edge state, and their localization along the nanoribbon structure of PbCF_2_. The edge states were obtained for a NR width of 64 Å. Since opposite edges present different equilibrium geometry, there is no mirror symmetry along the NR structure, the Dirac-like edge states exhibit a slight energy splitting at the Dirac crossing points. Similar Dirac-like linear energy dispersion, for the same NR width, was verified of the other PBXF_2_ systems.

The spacial distribution of the Dirac-like states near the Γ-point, depicted in Fig. 7(c), shows that those states lie near PbCF_2_ edge sites, with a penetration length of few units of PbCF_2_ (edge) chains. The spin-momenta of those states are locked. In this case, considering the electronic states above the Dirac point, on the energy bands 1 and 2, near the Γ point with wave vectors parallel to the edge sites (Γ-K direction), *viz.*: 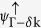 and 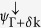, respectively, we find that they present opposite spin polarization; and thus, although lying on the same edge, backscattering processes are not allowed (
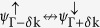
). The same happens with the energy bands, 3 (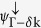) and 4 (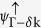), as well for the electronic states (near the Γ-point) below the Dirac crossing. This behavior is the main characteristic of the QSHE.

## Discussion

In summary, based on first principles calculations, we find that fluorinated binary compounds of 2D PbX, PbXF_2_ for X = C, Si, Ge, and Sn present topological nontrivial phase. The fluorination of PbX layers (PbX → PbXF_2_) remove the metallic *π* states, which is quite important step to get the TI phase. Indeed, the TI phase in PbXF_2_ was (firstly) verified by examining the topological invariant Z_2_, based upon the WCC approach; we obtained Z_2_ = 1 for all PbXF_2_ structures. Further electronic structure calculation revealed that such a 2D TI phase is ruled by a suitable synergy between the crystal field and SO interactions in PbXF_2_. Upon the formation of Pb–X chemical bonds, (i) the crystal field places antibonding orbital [|*S*_*Pb*_〉 − |*S*_*X*_〉]^−^ below the partially occupied |*P*_*x*,*y*_〉^+^ state; meanwhile (ii) the SO interaction remove its degeneracy, opening an energy gap (at the Γ-point) between the |*P*_*x*,*y*_〉^+^ orbitals. The nontrivial phase in PbXF_2_ is dictated by the reversed energy order between the antibonding *s* and the highest occupied *p* orbitals. In (i), we found that the energy splitting between the bonding and antibonding *s* orbitals is inversely proportional to the equilibrium bond length of PbXF_2_, while the energy gap in (ii) is proportional to the atomic number (Z) of the X element. Indeed, we found an energy gap of 0.99 eV at the Γ point, and an indirect band gap up to 0.77 eV in PbSnF_2_. Such a large band gap make these systems not only experimentally feasible to room temperature, but promising for applications in 2D topological devices. Finally, by considering nanoribbon structures, we verify the formation of 1D Dirac-like topologically protected states localized along the edge sites of PbXF_2_.

## Methods

The first-principles calculations were based on the density functional theory (DFT)^28^,^29^ as implemented on the OpenMX code^30^. For the exchange-correlation functional we used the GGA-PBE approximation^31^. The spin-orbit interaction was included via norm-conserving fully relativistic *j*-dependent pseudopotentials scheme, in the non-collinear spin-DFT formalism^32^. The electron wavefunctions are expanded as linear combinations of pseudo atomic orbitals (LCPAOs)^30^, which are generated bu using a confinement potential scheme^33^ with a cutoff radius of 8.0, 6.0, 7.0, 7.0, 7.0 and 6.0 for Pb, C, Si, Ge, Sn and F, respectively. In the self-consistent calculations of charge density for the unit cells a 15 × 15 × 1 Monkhorst-Pack k-grid is employed and for the nanoribbons we used 15 × 1 × 1 k-points in the periodic direction. All the systems investigated were fully relaxed until the Hellmann-Feynman the residual forces were smaller than 0.001 eV/Å. In all systems we used a vacuum distance of 20 Å in the non-periodic direction. For the phonon spectra we used the SIESTA code^34^ with a Duble Zeta plus a Polarization function for the basis set and all others criteria were keep the same as in the others calculations. In order to verify the topological character of the PbXF_2_ compounds, we determine the Z_2_ invariant based upon the the evolution of the Wannier Center of Charges (WCCs) method, as proposed by Soluyanov and Vanderbilt^35^,^36^. Here the calculation was performed by using a plane wave basis set as implemented in the VASP code^37^,^38^. The electronic band structure of the PbXF_2_ systems were calculated by using the standard GGA-PBE approach, as well the hybrid functional HSE-06 of Heyd, Scuseria, and Ernzerhof (HSE)^26^,^27^.

## Additional Information

**How to cite this article**: Padilha, J. E. *et al*. A new class of large band gap quantum spin hall insulators: 2D fluorinated group-IV binary compounds. *Sci. Rep.* **6**, 26123; doi: 10.1038/srep26123 (2016).

## Supplementary Material

Supplementary Information
