# Supplementary material for: A new class of large band gap quantum spin hall insulators: 2D fluorinated group-IV binary compounds
Source: Sci Rep. 2016 May 23;6:26123. doi: 10.1038/srep26123 (PMC4876509; doi:10.1038/srep26123)
Supplement: Supplementary Information [file srep26123-s1.pdf]

Supplementary Information for: A new class of large band gap quantum spin hall insulators: 2D fluorinated group-IV binary compounds

J. E. Padilha,[1] R. B. Pontes,[2] T. M. Schmidt,[3] R. H. Miwa,[3] and A. Fazzio[4,5]

[1] *Universidade Federal do Paraná, Campus Avançado de Jandaia do Sul, Jandaia do Sul, PR, Brazil*

[2] *Instituto de Física, Universidade Federal de Goiás, 74690-900, Goiânia, GO, Brazil*

[3] *Instituto de Física, Universidade Federal de Uberlândia, Uberlândia, MG, Brazil*

[4] *Centro de Ciências Naturais e Humanas, Universidade Federal do ABC, Santo André, São Paulo, Brazil 09210-170.*

[5] *Instituto de Física, Universidade de São Paulo, CP 66318, 05315-970, São Paulo, SP, Brazil.*

(Dated: February 24, 2016)

We have done further calculations by considering $PbCY_2$, $PbSiY_2$, $PbGeY_2$, and $PbSnY_2$, for Y = H, Cl, Br, and I; where we find that (i) the TI character has been suppressed in $PbXH_2$ (X = C, Si, Ge, abd Sn), while (ii) the other $PbCY_2$, $PbSiY_2$, $PbGeY_2$, and $PbSnY_2$ counterpart systems, for Y = Cl, Br, and I, are energetically less stable than $PbXF_2$.

HSE-06 BAND STRUCTURES

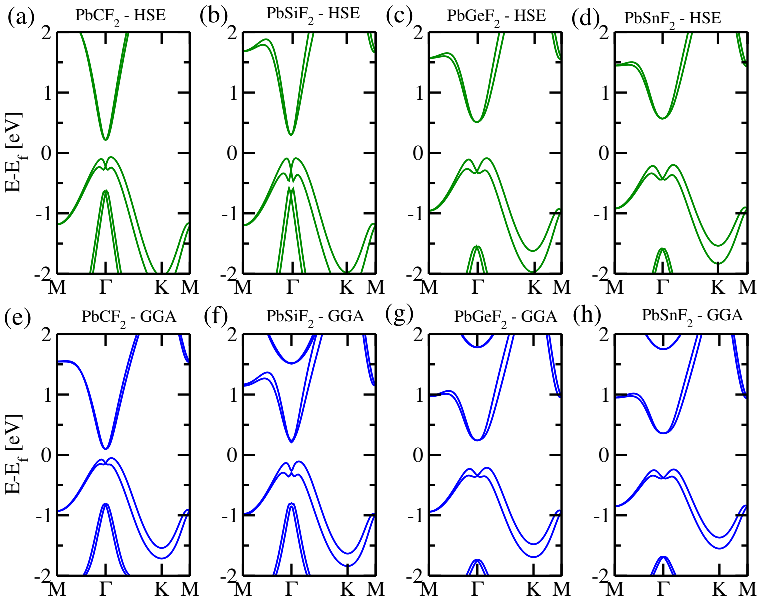

FIG. 1. (HSE-06 Band structures calculated for the (a) $PbCF_2$; (b) $PbSiF_2$; (c) $PbGeF_2$; (d) $PbSnF_2$. GGA-PBE Band structures calculated for the (a) $PbCF_2$; (b) $PbSiF_2$; (c) $PbGeF_2$; (d) $PbSnF_2$.
